# Ten-year trajectories of ultra-processed food intake and prospective associations with cardiovascular diseases and all-cause mortality: findings from the Whitehall II cohort study

**DOI:** 10.1186/s12937-025-01144-2

**Published:** 2025-05-11

**Authors:** Mengmei E. Wang, Clare H. LIewellyn, Michail Katsoulis, Tasnime N. Akbaraly, Samuel J. Dicken, Jiahao Liu, Adrian Brown, Annie Britton

**Affiliations:** 1 Research Department of Epidemiology and Public Health, University College London, London, UK; 2 Research Department of Behavioural Science and Health, University College London, London, UK; 3 MRC Unit for Lifelong Health and Ageing, University College London, London, UK; 4 Desbrest Institute of Epidemiology and Public Health, Université de Montpellier, Institut National de Santé et de Recherche Médicale (INSERM), Montpellier, France; 5 Centre for Obesity Research, Department of Medicine, University College London, London, UK; 6 Faculty of Medicine, Dentistry and Health Sciences, University of Melbourne, Melbourne, Australia; 7 Centre for Eye Research Australia, Royal Victorian Eye and Ear Hospital, Melbourne, Australia

**Keywords:** Epidemiology, Nutritional epidemiology, Food processing, Cardiovascular diseases, Ultra-processed foods, Public health, Dietary pattern, Food policy

## Abstract

**Background:**

Ultra-processed food (UPF) intake has been associated with adverse health outcomes; however, research on UPF intake and cardiovascular disease (CVD) prognosis has largely neglected its longitudinal pattern over time. This study investigated trajectories of UPF intake over a decade and their prospective associations with the risk of fatal and non-fatal CVD, as well as all-cause mortality, using data spanning from 16 to 19 years.

**Methods:**

This study utilized data from the British Whitehall II cohort study, including 7,138 participants (68.3% male; median baseline age 60.4 years), all free of CVD at baseline. Dietary intake was assessed using a validated 127-item food frequency questionnaire at three time points: phase 3 (1991–1994), phase 5 (1997–1999), and phase 7 (2002–2004). UPF intake was estimated using the Nova classification, and group-based trajectory modelling identified different longitudinal consumption patterns. Phase 7 (2002–2004) was the baseline for subsequent monitoring of cardiovascular events and mortality outcomes until 2019/2021. Multivariate Cox proportional hazards models were used to estimate the hazard ratios (HRs) and 95% confidence intervals (CIs), adjusting for socio-demographics, lifestyle, diet quality, energy intake, and clinical factors.

**Results:**

Three distinct UPF trajectory groups were identified: high (26.2% of participants), moderate (52.9%) and low UPF intake (20.9%). All groups showed a slight increase in UPF intake over time. Over the median follow-up of 16 years for incident cases and 19 years for mortality, we observed 1,128 incident CVD events, 859 CHD cases and 1,314 deaths. The highest vs. lowest UPF intake group had a 23% higher risk of CVD (HR 1.23, 95% CI 1.01 to 1.40), and a 32% higher risk of CHD (HR 1.32, 95% CI 1.06 to 1.65). No significant associations were observed between UPF trajectory groups and CVD mortality, CHD mortality, or all-cause mortality.

**Conclusions:**

Sustained high UPF intake over 10 years was associated with increased risks of non-fatal CVD and CHD but not with CVD-specific, CHD-specific, or all-cause mortality. These findings suggest that sustained high intake of UPF may be a modifiable risk factor for preventing non-fatal cardiovascular risks.

**Supplementary Information:**

The online version contains supplementary material available at 10.1186/s12937-025-01144-2.

## Introduction

Cardiovascular disease (CVD) is a leading cause of chronic disability and mortality worldwide [1]. Poor dietary behaviours represent a significant modifiable risk factor for CVD, making diet a critical focus for cardiovascular prevention strategies [2]. Over recent decades, global dietary patterns have shifted markedly from traditional, whole foods diets toward increased intake of ultra-processed foods (UPF). UPF, as most commonly defined by the Nova classification [3], are industrial reformulations produced using compounds extracted, derived, or synthesized from high-yield crops or by-products of intensive animal agriculture, resulting in highly modified products [3, 4]. Examples of UPF include breakfast cereals, processed meats, beverages that are either artificially or sugar-sweetened, plant-based meat substitutes, and plant-based milks (e.g., soya milk), as well as various ready-to-eat meals. Although some plant-based UPF and cereals may be viewed as healthier alternatives, many still contain excessive amounts of sodium, sugar, and/or saturated fats, along with non-nutritive additives and containments introduced during processing [5]. In the UK, for example, UPF accounts for 56.8% of adult total energy intake [6]. Compared to minimally processed foods, UPF is disproportionately higher in added sugars, sodium, saturated fats and trans fats, and refined carbohydrates, while typically being lower in protein and micronutrients [5]. While causal mechanisms are not confirmed, there are several hypothesised pathways through which UPF could compromise health: poorer nutrient profile; higher energy density; processing (e.g. food matrices degradation); packaging and use of additives [7].

Growing evidence from epidemiological studies has demonstrated associations between greater UPF intake and an increased risk of a wide range of cardiometabolic diseases, including obesity, type 2 diabetes, CVD, and metabolic dysfunction-associated steatotic liver disease (MASLD) [8]. Specifically, a meta-analysis of eleven cohort studies reported a 35% higher risk of developing cardiovascular events when comparing the highest versus the lowest UPF intake category [9]. Another meta-analysis of twenty studies demonstrated a smaller effect size, with a 1.9% increased risk of cardiovascular events for each additional daily serving of UPF [10]. Despite these findings, the evidence linking UPF intake with CVD incidence, as well as CVD and CHD mortality, remains limited [8]. Further high-quality research is needed to draw robust conclusions and clarify causal relationships.

Most studies estimating UPF intake have relied on a single time point, or a cumulative average of intake [11, 12, 13]. These approaches can yield heterogeneous results and have inherent limitations, as they treat UPF exposure as static over time. This assumption overlooks the potential for changes in UPF intake to influence health outcomes and fails to capture the shape of intake trajectories [14]. In contrast, trajectory modelling is increasingly recognized as a robust approach for integrating exposure data collected over extended periods. This method offers a valuable alternative for association studies by capturing distinct exposure patterns and accounting for potential cumulative effects [15]. To date, no studies have characterized distinct trajectories of UPF intake or examined their long-term associations with the risk of CVD and mortality. Given the significant burden of CVD and the ongoing debate regarding new policy measures to regulate UPF, rigorous evidence is needed to examine the longitudinal patterns of UPF intake and their impact on cardiovascular health. Therefore, the primary aim of this study was to characterize long-term trajectories of UPF intake over a decade. The secondary aim was to examine the associations between these trajectories and the risk of CVD and mortality outcomes. Repeated measures of UPF intake during midlife were analysed to investigate their prospective associations with incident CVD and subsequent mortality outcomes in participants from the UK Whitehall II cohort.

## Methods

### Study population

This study utilized data from the British Whitehall II cohort study, which invited all civil servants aged 35–55 years from 20 London-based departments to participate between 1985 and 1988. A total of 10,308 participants (73% response rate) were enrolled [16]. Follow-up clinical examinations were conducted approximately every five years during the following phases: phase 3 (1991–1994), phase 5 (1997–1999), phase 7 (2002–2004), phase 9 (2007–2009), phase 11 (2012–2013), phase 12 (2015–2016), and phase 13 (2019–2022). To develop the trajectory of UPF intake groups, we utilized dietary data from phases 3, 5, and 7, allowing us to fully capture longitudinal patterns of UPF intake. For our secondary aim, which examined associations between UPF trajectory groups and subsequent health outcomes, we designated phase 7 (2002–2004) as the ‘baseline.’ Participants with at least one measure of UPF intake at phases 3, 5, or 7 were initially considered (*n* = 8,577). Exclusion criteria included pre-existing CVD or history of CVD at or before phase 7, missing CVD follow-up data (*n* = 1,269). Participants with implausible total energy intake estimates (< 500 kcal or > 3,500 kcal for women and < 800 kcal or > 4,000 kcal for men) were also excluded (*n* = 170). The final analytic sample consisted of 7,138 participants. A detailed flowchart of the selection process is provided in Fig S1

### Dietary assessment and Estimation of UPF intake

Dietary intake was assessed at phases 3, 5, and 7 using a validated 127-item semi-quantitative food frequency questionnaire (FFQ). Validation of the FFQ within the Whitehall II cohort has been described in detail elsewhere [17]. Participants provided self-reported estimates of their average consumption of standard portion sizes for each food item over the past year. Frequency of consumption for each food was measured using a nine-point scale, ranging from “never or less than once per month” to “six or more times per day.” An example of the FFQ questionnaire is provided in Table S1. The selected frequency category for each food item was converted to a daily intake and linked to a corresponding nutrient database [18]. Daily food intake in grams was estimated by multiplying the frequency of consumption for each food item by its standard portion size. Nutrient intakes were calculated by multiplying the consumption frequency of each food item by its nutrient content and summing across all items. Total energy intake was then estimated from macronutrient values, as reported in previous studies [18]. The Nova classification was used to identify FFQ items that fall into the UPF category. Detailed information on the classification of the 127 FFQ items is provided in Table S2 and File S2.

The proportion of UPF was calculated in each participant’s diet by dividing the total amount of UPF consumed per day (in grams) by the total amount of all foods consumed per day (in grams) and then multiplying the resulting ratio by 100. This approach yields a percentage reflecting the share of an individual’s daily diet (by weight) that is composed of ultra-processed items. This method accounts for UPF items with minimal or no caloric content, such as artificially sweetened beverages, and incorporates non-nutritional aspects related to food processing [19].

### Assessment of covariates

Based on previous literature, covariates included socio-demographic factors, health behaviours, lifestyle factors, and clinical risk factors, all measured at baseline (phase 7: 2002–2004) through self-reported questionnaires or clinical examinations [16].

Socio-demographic variables included biological sex at birth, age (continuous), marital status (married/cohabiting or not), family history of CVD, CHD, and cancer; ethnicity (White or non-White); educational attainment (below secondary, secondary, or university level); and socio-economic status (SES) based on civil service employment grade: low (clerical/support), intermediate (professional/executive), or high (administrative) [16].

Behavioural covariates included smoking status (never smokers, ex-smokers, and current smokers); physical activity, categorized as “active” (> 2.5 h/week of moderate physical activity or > 1 h/week of vigorous physical activity), “inactive” (< 1 h/week of both moderate and vigorous physical activity), or “moderately active” (neither active nor inactive); alcohol consumption, categorized as low (no alcohol consumption in the past week), moderate (1–14 units/week), or heavy (≥ 15 units/week) [20, 21].

Total energy intake (in kilocalories) was included as a continuous variable. Nutritional indicators of diet quality included daily intakes of sodium (mg/day), fat (g/day), and sugar (g/day). Clinical factors considered were as follows: body mass index (BMI) was calculated as weight (kg) divided by height (m) squared (kg/m²) and treated as a continuous variable. Hypertension was defined as either systolic blood pressure ≥ 140 mmHg, diastolic blood pressure ≥ 90 mmHg, or the use of antihypertensive medication. Prevalent Type II diabetes was defined as HbA1c ≥ 6.5% (48 mmol/mol), self-reported doctor-diagnosed diabetes, use of diabetes medication, or records from hospitalizations. Dyslipidaemia was defined as meeting any one of the following criteria: total cholesterol > 6.0 mmol/L; triglycerides > 1.7 mmol/L; HDL-cholesterol < 1.0 mmol/L; LDL-cholesterol > 4.0 mmol/L; or receiving lipid-lowering medication [22].

### Outcome ascertainment

The primary outcomes of this study were incident cases of CVD and CHD. Outcome data were sourced from the National Health Service (NHS) Hospital Episode Statistics database and mortality registers, linked via individual NHS identification numbers. Diagnoses were coded according to the International Classification of Diseases, 9th and 10th Revisions (ICD-9 and ICD-10), and were recorded up to October 2, 2019 [23].

Secondary outcomes included CVD mortality, CHD mortality, and all-cause mortality. Participants were linked to national mortality registers via their unique identification numbers. Data on deaths were sourced from NHS Digital, recorded up to 28th February 2021. A detailed classification of CVD, CHD, and mortality cases is provided in Table S3.

### Statistical analyses

Descriptive analyses were performed to summarize baseline (phase 7) sample characteristics. Continuous variables were presented as medians with interquartile ranges (IQR), while categorical variables were reported as counts and percentages. Chi-square tests were used to compare categorical variables across UPF intake trajectory groups, and Kruskal–Wallis tests were applied to assess differences in continuous variables across these groups.

### Multiple imputation of missing covariates

Before performing inferential analyses, missing data for covariates were addressed by using multiple imputation using random forest algorithm. Education had the highest proportion of missing data (23.9%). A random forest algorithm imputation method was used, generating five imputed datasets over 50 iterations to ensure convergence. Random forest was selected for its capability to model complex interactions and nonlinear relationships, thereby improving the accuracy of imputations for both continuous and categorical variables [24].

### Creating trajectories of UPF intake

Group Based Trajectory Modelling (GBTM) was applied to determine groups with similar UPF intake trajectories using the *traj* command in STATA [25]. GBTM is particularly suited for capturing heterogeneous trajectories in populations where a single set of parameters cannot adequately represent variability in patterns of behaviour [15]. The optimal number of trajectory groups was determined by testing models with 2 to 5 groups and evaluating them against goodness-of-fit criteria, including the Bayesian Information Criterion (BIC), log Bayes Factors, a minimum group size of 5% of participants, consistency between estimated and actual group membership proportions, odds of correct classification greater than 5, and average posterior probabilities (AvePP) above 0.70 as an indicator of high classification accuracy [25].

The longitudinal UPF intake data were modelled using a censored normal model, accounting for the effects of outliers, which is suitable for continuous data. Time-varying covariates from phases 3, 5, and 7, including sociodemographic, lifestyle, indicators of diet quality, and clinical factors, were incorporated to capture dynamic influences and improve trajectory accuracy. A quadratic trajectory model with three distinct UPF intake trajectories provided the best fit to the data and provided sufficient classification accuracy, with AvePP ranged from 0.78 to 0.85. Each participant was subsequently assigned to the corresponding trajectory group according to the maximum likelihood estimation. Results from the model selection process are provided Table S5 and S6 .

### Survival analysis on the associations between UPF trajectories and outcomes

Cox proportional hazards models were used to assess the prospective associations between the three UPF intake trajectories and: (i) incident cases of CVD, CHD; and (ii) CVD mortality, CHD mortality, and all-cause mortality. Follow-up time was measured from the date of the baseline (phase 7) questionnaire to the occurrence of an event, death, or the end of the follow-up period, whichever occurred first. Hazard ratios (HR) and 95% confidence intervals (CI) according to each trajectory group were obtained. Five models were analysed: (i) adjusted for sex, age at baseline (phase 7), and ethnicity; (ii) further adjusted for SES, marital status, education, physical activity, alcohol consumption status, smoking status, family history of CVD, CHD and cancer; (iii) additionally adjusted for total energy intake; and (iv) further adjusted for indicators of diet quality, including total sugar, total sodium, and total fat intake; (v) final additional adjustment of BMI, hypertension, prevalent type II diabetes, and dyslipidaemia.

A series of sensitivity analyses was conducted to enhance the robustness of the study. First, we repeated the Cox analyses among (i) participants with complete UPF intake data across all three phases. And (ii) participants with no missing data on both UPF intake and covariates, using the fully adjusted Model 5. Our primary analysis employed GBTM to characterize patterns of UPF intake across phases 3, 5, and 7. Although this method provided valuable insights into long-term consumption trajectories, it may not fully capture the variability among individuals who experience pronounced changes in UPF intake over a decade that could influence cardiovascular outcomes. Therefore, we conducted an exploratory ad hoc analysis to identify participants with substantial changes in UPF intake from phase 3 (1991–1994) and phase 7 (2002–2004) and their prospective association with cardiovascular outcomes. We derived the 33rd and 66th percentiles of UPF intake from phase 3 data to establish fixed cut-points for low, moderate, and high consumption, and applied these same thresholds to the phase 7 UPF data to ensure classification relative to the baseline distribution. Participants were then categorized into five groups: those consistently in the lowest tertile (low), those consistently in the highest tertile (high), those shifting from the lowest tertile at phase 3 to the highest at phase 7 (low to high), those shifting from the highest tertile at phase 3 to the lowest at phase 7 (high to low), and those with stable moderate intake as well as individuals transitioning from low to moderate or high to moderate (moderate). This analysis allowed us to assess whether participants with substantial changes in UPF intake over the study period were differentially associated with cardiovascular outcomes, thereby capturing dynamic shifts not fully represented by the trajectory-based approach.

The proportional hazards assumption was assessed using Schoenfeld residuals tests, no violation was detected (all p-values > 0.05; Fig S2).

Statistical analyses were performed using STATA version 18.0 (StataCorp, College Station, TX) and R Studio Desktop version 4.3.0. Imputation was performed using “mice” packages using R. Statistical significance was set at *p* < 0.05.

## Results

### Participants’ characteristics by trajectory groups

**Fig. 1:**
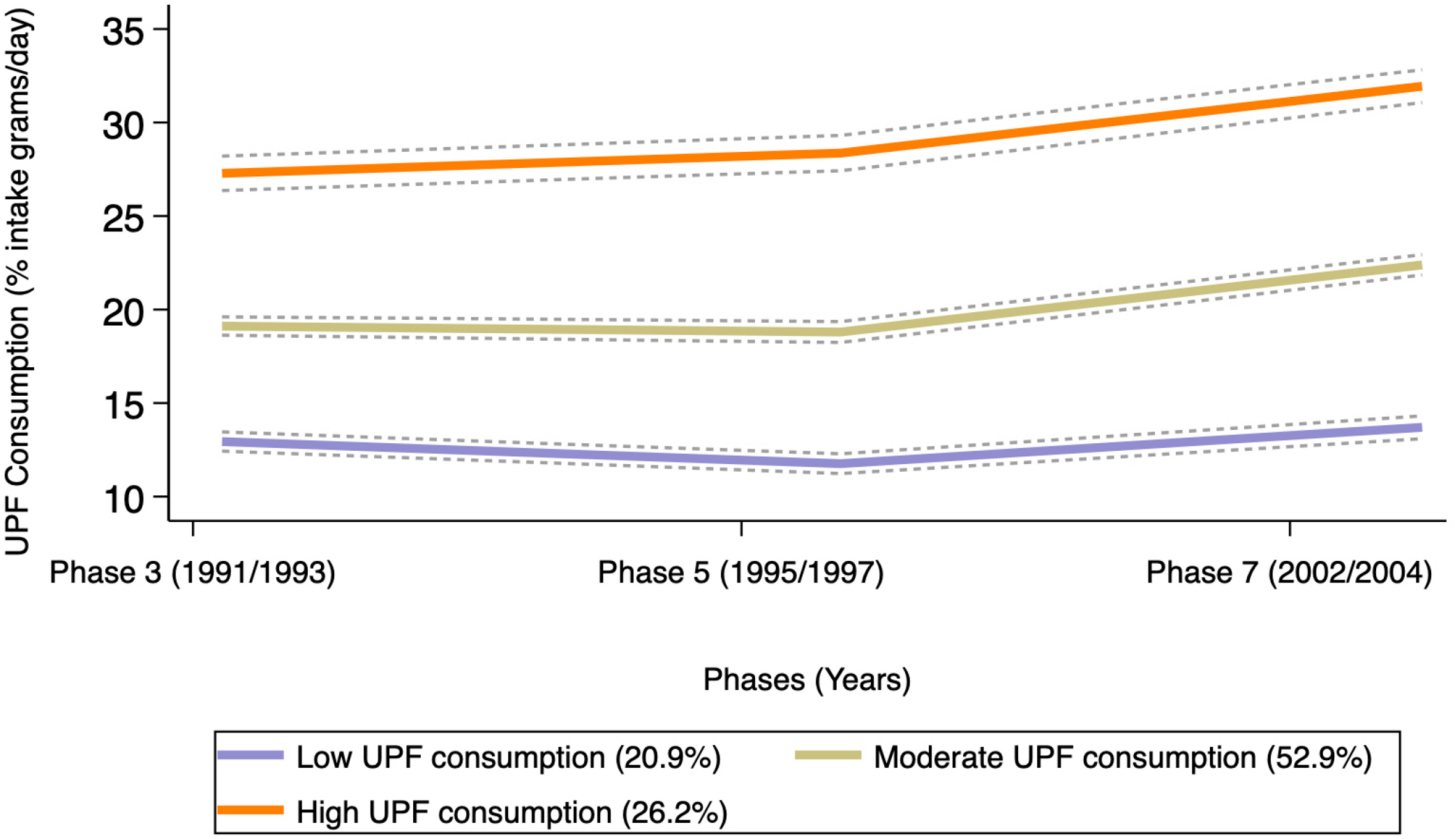
Group-based trajectory modelling derived UPF consumption trajectories and 95% CI starting from phase 3 (1991–1994) to phase 7 (2002–2004) (*n* = 7,138)

Among the 7,138 participants over a span of 10 years, three UPF intake trajectory groups were identified: low UPF intake (20.9% of participants); moderate UPF intake (52.9%); and high UPF intake (26.2%). All three groups maintained relatively stable UPF intake, with a slight increase around age 50 (Fig**.** 1).

Baseline characteristics for each trajectory group are reported in Table 1. Participants in the high UPF intake group were more likely to be physically inactive, less likely to be heavy drinkers, and more likely to have lower educational attainment and employment grade than those in the low UPF intake group. Additionally, compared with participants who had low UPF intake, the high UPF intake group exhibited poorer diet quality and health profiles. They had higher intakes of sodium, fat, sugar, and total energy, along with a greater rate of obesity, type II diabetes, hypertension, and dyslipidaemia.

**Table 1:** Participants characteristics (Phase 7: 2002/2003) for the overall participants and by ultra-processed food trajectory groups^a^

	All participants (*n* = 7,138)	Low UPF intake (*n* = 1808)	Moderate UPF intake (*n* = 4232)	High UPF intake (*n* = 1098)	*P* value
Cardiovascular diseases					
No. of cases	1128	271	666	191	
Coronary heart diseases					
No. of cases	859	200	505	154	
CVD mortality					
No. of cases	284	66	176	42	
CHD mortality					
No. of cases	124	28	75	21	
All-cause mortality					
No. of cases	1314	781	356	177	
Age, median (IQR), years	60.4(56.1–66.5)	61.0(56.0-67.1)	61.0(56.0-66.2)	60.2(56.0-66.4)	0.001
Sex, n (%)					
Male Female	4870 (68.3) 2268 (31.7)	1225 (67.7) 583 (32.3)	2907 (68.7) 1325 (31.3)	746 (68.0) 352 (32.0)	0.03
Ethnicity, n(%)					0.02
White Non- White	6544 (91.7) 594 (8.3)	1626 (89.9) 182 (10.1)	3890 (91.9) 342 (8.1)	1028 (93.6) 70 (6.4)	
Family history of CVD, CHD, n(%) No Yes	6447(90.3) 691 (9.7)	1620(89.6) 188 (10.4)	3826 (90.4) 406 (9.6)	1001 (91.2) 97 (8.8)	0.37
Family history of cancer, n(%) No Yes	3449 (48.3) 3689 (51.7)	864 (47.8) 944 (52.2)	2038 (48.2) 2194 (51.8)	547 (49.8) 551 (50.2)	0.54
Physical activity, n(%)					0.007
Inactive Active Moderate active	385(5.4) 6319(88.5) 434(6.1)	88 (4.9) 1616 (89.3) 104 (5.8)	222 (16.2) 3767 (78.5) 243 (5.3)	75 (6.8) 936 (85.3) 87 (7.9)	
Alcohol consumption, n(%)					< 0.001
Abstainers Moderate drinkers Heavy drinkers	1557 (21.8) 3516 (49.3) 2065 (28.9)	382 (21.1) 792 (43.8) 634 (35.1)	877 (20.7) 2154 (50.9) 1201 (28.4)	298 (27.0) 570 (52.0) 230 (21.0)	
Smoking, n(%)					0.216
Never smoker Ex-smoker Current smoker	3444 (48.2) 3140 (44.0) 554 (7.8)	863 (47.7) 807 (44.6) 138 (7.6)	2060 (48.6) 1860 (44.0) 312 (7.4)	521 (47.4) 473 (43.1) 104 (9.5)	
Employment grade, n(%)					0.001
Low (clerical or support roles) Intermediate (professional roles) High (administrative roles)	818 (11.5) 3130 (43.8) 3190 (44.7)	211 (11.7) 779 (43.1) 818 (45.2)	462 (10.9) 1827 (43.2) 1943 (45.9)	145 (13.2) 524 (47.7) 429 (39.1)	
Education, n(%)					0.002
< Secondary Secondary University	2636 (36.9) 1919 (26.9) 2583 (36.2)	643 (35.6) 454 (25.1) 711 (39.3)	1554 (36.7) 1154 (27.3) 1524 (36.0)	439 (40.0) 311 (28.3) 348 (31.7)	
Hypertension, n(%)					0.12
Yes No	1787 (25.0) 5351 (75.0)	465 (25.7) 1343 (74.3)	1074 (25.4) 3158 (74.6)	248 (25.8) 850 (74.2)	
Type II diabetes, n(%)					0.40
Yes No	245 (3.4) 6893 (96.6)	62 (3.4) 1746 (96.6)	138 (3.3) 4094 (96.7)	45 (4.1) 1053 (95.9)	
Dyslipidaemia, n(%)					0.0001
Yes	1903 (26.7)	411 (21.6)	1162 (27.5)	330 (30.0)	
No	5235 (73.3)	1397 (77.3)	3070 (72.5)	768 (70.0)	
BMI, median (IQR), (kg/m^2^)	26.1(24.0-28.6)	25.9(23.8–28.2)	26.2(24.1–28.7)	26.4(24.2–29.3)	0.15
Total energy intake, median (IQR), (kcal/day)	2067(1716–2448)	1980(1664–2339)	2101(1739–2473)	2075(1737–2525)	0.0001
Total fat intake, median (IQR), (g/day)	72.8(56.4–92.1)	66.3(61.9–86.9)	74.3(58.1–93.5)	75.1(59.9–94.0)	0.0001
Total sugar intake, median (IQR), (g/day)	126(99.2–158)	122(94.9–156)	127(100–159)	127(101–162)	0.0001
Total sodium intake, median (IQR), (mg/day)	2537(1974–3161)	2356(1818–2962)	2590(2040–3210)	2626(2061–3256)	0.0001

**Abbreviations**: CVD, cardiovascular diseases; CHD, coronary heart diseases; CMD, cardiometabolic disease; BMI, body mass index

^a^ Continuous variables are expressed as median (IQR), and categorical variables as n (%)

^b^ P values were calculated using χ2 test for categorical variables or analysis of variance (Kruskal–Wallis test) for continuous variables

### Associations between ultra-processed food intake and cardiovascular diseases

Over a median follow-up of 16 years (114,208 person-years), 1,128 incident CVD events were recorded: 24.0% in UPF trajectory group 1 (low UPF intake), 59.0% in group 2 (moderate UPF intake), and 17.0% in group 3 (high UPF intake). A total of 859 incident CHD events were observed, with 23.3%, 58.8%, and 17.9% occurring in trajectory groups 1, 2, and 3, respectively. Over a median follow-up of 19 years, there were 1,314 deaths, including 284 from CVD and 124 from CHD.

**Fig. 2:**
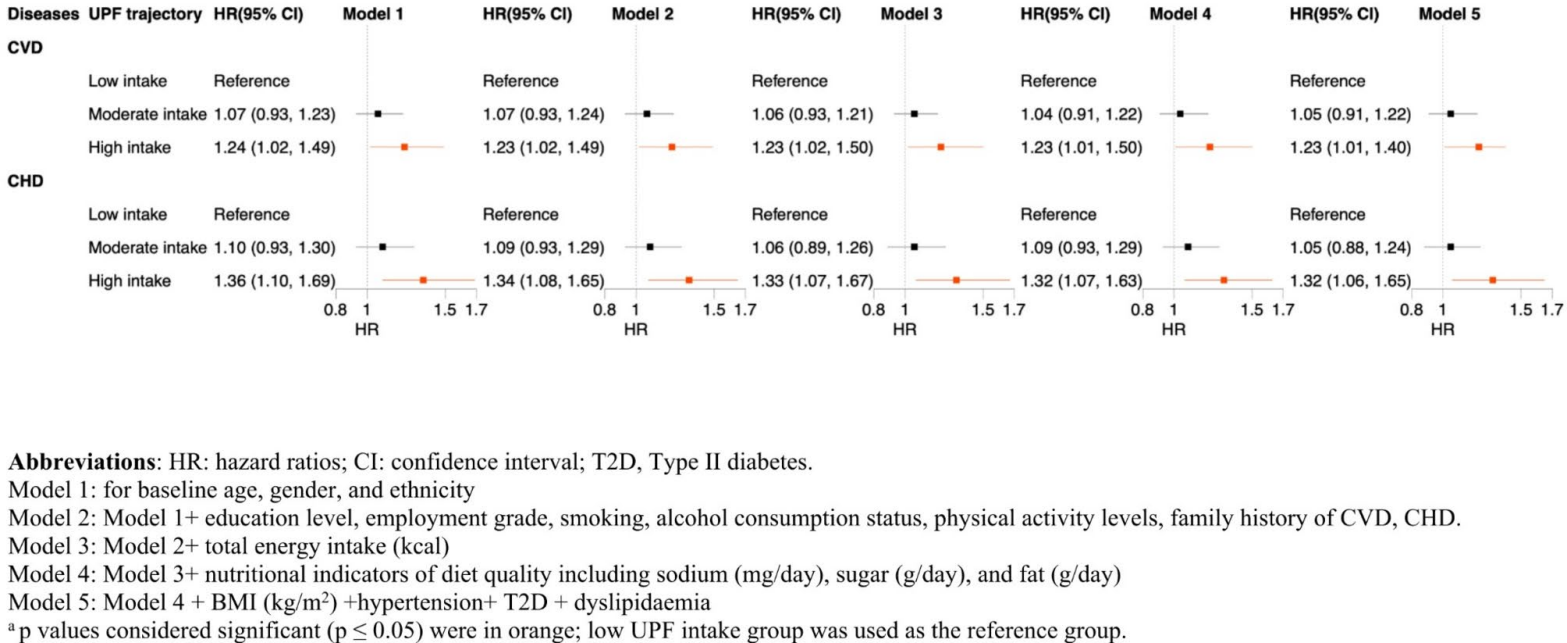
Prospective associations between trajectories of ultra-processed food intake and risk of cardiovascular and coronary heart diseases in the Whitehall II cohort (2004–2019, *n* = 7,138) from multivariable Cox proportional hazards models^a^

Fig**.** 2 illustrates the associations between UPF trajectories and the incidence of CVD and CHD. Participants in the high UPF intake trajectory had a significantly increased risk of both CVD and CHD compared to those in the low intake trajectory. After adjustment for sociodemographic and lifestyle factors, participants in the high UPF intake group had a 23% increased risk of CVD (HR 1.23, 95% CI 1.02 to 1.49) and a 34% increased risk of CHD (HR 1.34, 95% CI 1.08 to 1.65) compared to those in the low UPF intake group. These associations remained largely unchanged after further adjustment for total energy intake and diet quality proxies (HR 1.23, 95% CI 1.01 to 1.50 for CVD and HR 1.32, 95% CI 1.07 to 1.63 for CHD). Additional adjustment for BMI, hypertension, prevalent type II diabetes and dyslipidaemia did not materially alter the effect sizes (HR 1.23, 95% CI 1.01 to 1.40 for CVD and HR 1.32, 95% CI: 1.06 to 1.65 for CHD). Across all models, the moderate UPF intake group did not exhibit a significantly higher risk of CVD or CHD compared to the low intake group, although HRs were consistently higher.

**Fig. 3:**
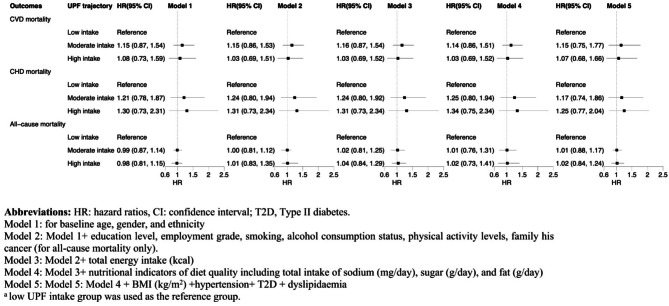
Prospective associations between trajectories of ultra-processed food intake and risks of cardiovascular mortality, coronary heart disease mortality, and all-cause mortality in the Whitehall II cohort (2004–2021, *n* = 7,138) from multivariable Cox proportional hazards models^a^

No significant associations were observed between UPF intake trajectory groups and the risk of CVD mortality (moderate UPF intake: HR 1.15, 95% CI 0.75 to 1.77; high UPF intake: HR 1.07, 95% CI 0.68 to 1.66), CHD mortality (moderate UPF intake: HR 1.17, 95% CI 0.74 to 1.86; high UPF intake: HR 1.25, 95% CI 0.77 to 2.04), or all-cause mortality (moderate UPF intake: HR 1.01, 95% CI 0.88 to 1.17; high UPF intake: HR 1.02, 95% CI 0.84 to 1.24) (Fig**.** 3).

The results of the sensitivity analyses are presented in Tables S7 and S8. The findings remained consistent across two separate tests: one restricted to participants with complete UPF intake data across all three phases and another restricted to those with no missing data on both UPF intake and covariates.

### Ad- hoc analyses

Among the 7,138 participants, 6.7% were classified as ‘UPF low-to-high intake,’ with mean UPF intake increasing from 11.6 ± 2.8% (% g/day) at phase 3 to 34.4 ± 7.2% (% g/day) at phase 7. Another 12.3% were classified as ‘UPF high-to-low intake,’ with mean UPF intake decreasing from 28.5% ± 7.4(% g/day) to 12.5%± 3.4 (% g/day). The remaining participants were categorized as ‘consistent high UPF intake’ (24.6%), ‘consistent low UPF intake’ (16.1%), or ‘moderate UPF intake’ (40.3%). Among these five groups, only the “consistent high UPF intake” category was significantly associated with increased risk of CVD (HR 1.14, 95% CI 1.02–1.30) and CHD (HR 1.18, 95% CI 1.03–1.38) compared to the “consistent low UPF intake” category (Table S9). The “low-to-high” UPF intake group did not show a statistically significant increase in any outcome, although their HRs were consistently elevated with respect to both CVD and CHD.

## Discussion

In this large, prospective, population-based cohort study of 7,138 midlife adults, three distinct trajectories of UPF intake were identified over approximately 10-year period using group-based trajectory modelling. These trajectories were categorized as ‘low UPF intake,’ ‘moderate UPF intake,’ and ‘high UPF intake.’ A trajectory of sustained high UPF intake from midlife to early old age was associated with a 23% increased risk of incident CVD and a 32% increased risk of CHD over a median follow-up period of 16 years, after adjusting for socio-demographic factors, health behaviors, lifestyle factors, total energy intake, nutritional indicators of diet quality and clinical factors. No significant associations were observed between UPF trajectory groups and CVD- or CHD-related mortality, or all-cause mortality.

### Findings in context

Our findings are similar to previous studies that examined the association between UPF intake and CVD in non-clinical cohorts of adults during midlife with similar sociodemographic characteristics, suggesting that higher intake of UPF is associated with an increased risk of CVD in the general population [12, 26, 27, 28]. Of the 22 cohort studies reviewed in the systematic review [10], only five reported no significant association between UPF intake and the risk of CVD or CHD [29, 30, 31, 32, 33]. Our findings align with the majority, indicating a positive association, with effect sizes for CVD and CHD risk comparable to, or slightly lower than, those reported in studies from France, Sweden, the UK, and the US [11, 12, 26, 27, 28, 34, 35]. These studies typically estimated UPF intake using either the average from multiple 24-hour dietary records, baseline FFQ data, or single 24-hour dietary recall at one time point, with sample sizes ranging from 1,100 to 25,000. Notably, several studies reported a significant association with increased CVD risk only in the highest quintile or quartile of UPF consumption, consistent with the effect sizes observed in our highest UPF trajectory group [11, 34, 36]. Although direct comparisons are challenging due to differences in UPF intake categorization (e.g., quintiles or quartiles vs. trajectory groups), a consistent pattern emerges: high UPF intake is associated with increased CVD and CHD risk across varied methodologies and population samples.

Given the rising intake of UPF, our findings reflect the recent UPF intake patterns and their association with CVD risk. In line with previous studies, the proportion of UPF in the diet in different trajectory groups varied according to a person’s socioeconomic background and lifestyle [14, 37]. These results also highlight the social inequalities in food choices, as UPF is widely recognized for their affordability, convenience, and extended shelf life.

While existing evidence consistently suggests a link between UPF intake and increased risk of CVD or CHD [10], findings on CVD/CHD mortality and all-cause mortality have been inconsistent [9]. Several longitudinal studies have reported no significant association between UPF intake and risk of CVD mortality [38, 39, 40, 41], while other cohort studies have consistently found the association between UPF intake and risk of CVD mortality or all-cause mortality. One possible explanation for this discrepancy is in this cohort, high UPF consumers were less likely to be heavy drinkers, which may have contributed to the lack of association observed. As heavy drinking is a well-established risk factor for CVD, CHD, and all-cause mortality [42] and its lower prevalence among high UPF consumers may have counterbalanced the potential risks associated with UPF intake. Furthermore, discrepancies between studies may arise due to methodological differences, such as variations in the accuracy of cause-specific mortality data, limited statistical power due to small numbers of deaths, differences in the assessment and classification of UPF intake, and variations in sociodemographic, cultural, and lifestyle characteristics of study populations.

### Potential mechanisms of UPF associated with risk of CVD

Multiple mechanisms may underlie the association between sustained high UPF intake and CVD. Diets rich in UPF are generally of lower nutritional quality and often contain excessive salt, added sugars, and fats, all of which are established risk factors for CVD [7, 43]. In addition, these diets tend to correlate inversely with healthy eating patterns, which can further contribute to cardiovascular risks [2]. However, in our study, the association between high UPF intake group and risk of CVD and CHD was not attenuated much after adjustment for nutritional indicators of diet quality including total sugar, fat and sodium intake. While this suggests that the association might be independent of the nutritional profile of UPF, it could also reflect residual confounding. It has also been hypothesised that as UPF is typically energy dense, it encourages faster eating and excess energy intake, leading to obesity and increased CVD risk [44, 45]. In this study, the associations between UPF intake and the risks of CVD and CHD were attenuated but remained significant after adjusting for total energy intake. Further adjustment for BMI and prevalent clinical risk factors had minimal impact on the effect sizes for UPF intake associations with CVD and CHD risk, and subsequent mortality outcomes, with attenuation of less than 10%, suggesting that these associations may be independent of pre-existing metabolic conditions.

Lastly, the persistent association observed in the fully adjusted model between sustained high UPF intake and increased risk of CVD and CHD suggests that additional factors may be involved. These factors may include the degree of processing, the presence of additives, and chemicals from food packaging, all of which could contribute to inflammation, metabolic disturbances, and elevated CVD risk [7].

### Strengths and limitations

This study has several strengths. To the best of our knowledge, this is the first study to apply trajectory modeling to investigate repeated measures of UPF intake among midlife to older adults in the UK. Although the UPF intake trajectory groups visually appear stable over time, the patterns identified through the longitudinal repeated measures of UPF intake remain unique. Three aspects of the analytical strategy stand out: the combined use of trajectory modeling and Cox regression to identify distinct UPF intake patterns and their associations with CVD and mortality risk; the consideration of a comprehensive range of covariates, including demographics, health behaviors, total energy intake, nutritional indicators, and clinical factors, reduces the likelihood that results are driven by specific health profiles; and the potential to capture long-term dietary changes and their mechanisms through repeated measures of UPF intake. Leveraging repeated measures of UPF intake over a decade, alongside rigorous ascertainment of causes of death and CVD, CHD and mortality outcomes over 16 to 19 years of follow-up, our study demonstrated that both the accumulation and variation in UPF intake are important for a comprehensive understanding of the risk of developing new onset CVD, specifically, our study improves the understanding of the individuals with sustained high UPF intake experienced higher risk of CVD and CHD.

Several limitations should also be noted. Firstly, it is not possible to infer causality from this observational study. Despite extensive adjustments for socio-demographic factors, lifestyle behaviours, total energy intake, diet quality indicators, and clinical factors, the possibility of residual confounding cannot be ruled out. The Nova classification system also has its limitation as it does not account for detailed information on ingredients, cooking methods or brand names of packaged foods. Although other food classification systems exist [46], they are not applicable to Whitehall II FFQ. Additionally, dietary data were obtained from a self-reported FFQ, which may introduce recall bias and misclassification of UPF intake. However, study shows good agreement with using an FFQ to estimate UPF intake compared with 24-hour recalls [47]. Although some uncertainty in individual trajectory group membership may exist within the GBTM model, an ad hoc analysis, in which participants were reclassified based on tertile cut-off points, identified similar UPF intake patterns and yielded consistent results (Table S9), suggesting that such uncertainty is unlikely to significantly affect the resulting profiles of characteristics from GBTM model [15]. While our imputation covariates showed good alignment with the complete data (Table S4), we acknowledge that missing data may not be entirely missing at random, which could introduce bias. However, complete-case analyses resulted in similar results (Table S8). Furthermore, selection bias may have influenced our results, as individuals who followed a certain UPF trajectory may have developed the outcome prior to phase 7 (baseline) and were therefore excluded from the analyses. It is unavoidable that the small number of CVD and CHD mortality cases may have limited statistical power, reducing our ability to detect true associations with high UPF intake. Lastly, the study population comprised midlife British civil servants, which may limit the generalizability of the findings to populations with different socio-economic and ethnic backgrounds.

### Implications for policy and practice, and future research

This research provides valuable insights into the long-term risk of CVD associated with variations in sustained UPF consumption patterns. Given the ongoing debate among policymakers worldwide regarding the regulation of UPF in the food market [48, 49], these findings are timely to inform policymakers in implementing measures to reduce UPF intake, such as introducing clearer labeling, imposing marketing restrictions, and updating dietary guidelines to address food processing. Further research is needed to clarify causality and underlying mechanisms, and employing diverse study designs is essential for strengthening inferences. In settings where randomized controlled trials are impractical, advanced causal inference methods, such as target trial emulation [50] can help approximate randomization in observational data. These approaches may reveal pathways such as metabolic or inflammatory processes through which UPF influences CVD risk, thereby supporting targeted dietary recommendations and interventions.

## Conclusion

Our analysis of a cohort of midlife adults in the UK found that a sustained high intake of UPF over a decade was significantly associated with an increased risk of CVD and CHD over a 16-year follow-up period. The longitudinal trajectories of UPF intake identified in this study contribute to the growing body of epidemiological evidence that highlights the adverse effects of sustained high UPF intake on cardiovascular health outcomes. These findings emphasize the importance for regulating UPF in the food market and advising dietary intake. Future research is essential to elucidate the underlying mechanisms and strengthen the causal evidence linking UPF intake with cardiovascular diseases, with the aim of better informing policymakers and the public in this complex domain.

## Electronic supplementary material

Below is the link to the electronic supplementary material.

Supplementary Material 1

Supplementary Material 2

## Data Availability

Whitehall II data, protocols, and other metadata are available to the scientific community. Please refer to the Whitehall II data sharing policy at https://www.ucl.ac.uk/psychiatry/research/mental-health-older-people/whitehall-ii/data-sharing.

## Abbreviations

UPF: Ultra-processed food
CVD: Cardiovascular diseases
CHD: Coronary Heart Diseases
T2D: Type II diabetes
MASLD: Metabolic Dysfunction-associated Steatotic Liver Disease
FFQ: Food Frequency Questionnaire
NHS: National Health Service
ICD: International Classification of Diseases
GBTM: Group-based Trajectory Modeling
BIC: Bayesian Information Criterion
BMI: Body Mass Index
SD: Standard Deviation
HR: Hazard Ratio
CI: Confidence Interval
