# Supplementary material for: Ten-year trajectories of ultra-processed food intake and prospective associations with cardiovascular diseases and all-cause mortality: findings from the Whitehall II cohort study
Source: Nutr J. 2025 May 11;24:79. doi: 10.1186/s12937-025-01144-2 (PMC12067948; doi:10.1186/s12937-025-01144-2)
Supplement: Supplementary file 2 — Supplementary Material 2 [file 12937_2025_1144_MOESM2_ESM.docx]

**Supporting information File 1**

S1 Table. Example of Food Frequency Questionnaire administrated in Whitehall II cohort study^a^

S2 Table. Classification of the food items from the Whitehall II food frequency questionnaire according to the Nova system ^a^

S3 Table. ICD-9 and ICD-10 Codes for Cardiovascular Diseases, Coronary Heart Diseases, Mortality Outcomes, and All-Cause Mortality

S4 Table. Distribution of variables with missing data before and after multiple imputation

S5 Table. Model search process for Ultra-processed food Trajectories (n=7,318), UK Whitehall II study, 1991/1994-2002/2003

S6 Table. Determining the Highest model function of the 3 UPF trajectory groups (n=7,138)

S7 Table. Sensitivity analysis for association between UPF trajectories and risk of CVD, CHD and its subsequent all-cause mortality restricting to participants with all 3 UPF measures (N=4050)

S8 Table. Sensitivity analysis for association between UPF trajectories and risk of CVD, CHD and its subsequent all-cause mortality for complete case data only(N=3735)

S9 Table. Sensitivity analysis for association between 11-year change in UPF intake between phase 3 (1991/1994) and phase 7 (2002/2004) and risk of CVD, CHD and its subsequent all-cause mortality (N=7,138)

S1 Fig. Flowchart of sample selection

S2 Fig. Schoenfeld residuals

Fig S2a). Schoenfeld residuals for incident cardiovascular diseases

Fig S2b). Schoenfeld residuals for incident coronary heart diseases

Fig S2c). Schoenfeld residuals for cardiovascular diseases mortality

Fig S2d). Schoenfeld residuals for coronary heart disease mortality

Fig S2e). Schoenfeld residuals for all-cause mortality

S3 Fig. Longitudinal trends in UPF intake tertile across phase 3 (1991-1994) to phase 7 (2002-2004)

S1 Table. Example of Food Frequency Questionnaire administrated in Whitehall II cohort study^a^

| **Food Item** | **Standard Portion** | **Frequency Categories (Average Use Over Past 12 Months)** |
| --- | --- | --- |
| White bread and rolls | One slice or roll | Never or <1/month, 1–3/month, Once/week, 2–4/week, 5–6/week, Once/day, 2–3/day, 4–5/day, 6+/day |
| Chips or French fries | Medium serving | Never or <1/month, 1–3/month, Once/week, 2–4/week, 5–6/week, Once/day, 2–3/day, 4–5/day, 6+/day |
| Strawberries, raspberries | Medium serving (in season) | Never or <1/month, 1–3/month, Once/week, 2–4/week, 5–6/week, Once/day, 2–3/day, 4–5/day, 6+/day |

^a^For the full Whitehall FFQ, please refer to

<https://www.ucl.ac.uk/psychiatry/research/mental-health-older-people/whitehall-ii/data-collection/questionnaires>

The selected frequency category for each food item was converted to a daily intake using the following standard conversion factors: 0.033 (never or < once per month), 0.066 (1–3 times per month), 0.14 (once per week), 0.43 (2–4 times per week), 0.79 (5–6 times per week), 1 (once per day), 2.5 (2–3 times per day), 4.5 (4–5 times per day), and 6 (six or more times per day).

S2 Table. Classification of the food items from the Whitehall II food frequency questionnaire according to the Nova system ^a^

| Group 1  Unprocessed or Minimally Processed foods | Beef, Pork, Lamb, Chicken, White Fish, Roasted Potato, Oily Fish, Shellfish, Porridge, Boiled Potato, White Rice, Brown Rice, Pasta, Whole Meal Pasta, Eggs, Nuts, Tea, Coffee, De-Café, Fruit Juice, Apples, Pears, Oranges, Grapefruit, Bananas, Grapes, Melon, Peaches, Strawberry, Dried Fruit, Carrots, Spinach, Broccoli, Spring Greens, Brussel Sprouts, Cabbage, Mushroom, Peppers, Leeks, Onions, Cauliflower, Parsnip, Peas, Beans, Marrow, Garlic, Tomatoes, Full Cream Milk, Semi-Skimmed Milk, Skimmed Milk, Sterilized Milk, Channel Island Milk, Dried Milk, Green Salad, Lentils, Yoghurt |
| --- | --- |
| Group 2  Processed culinary ingredients | Single cream, Double cream, Butter, Sugar |
| Group 3  Processed foods | Potato salad, Peanut butter, Wine, Beer, Tinned fruit, Cheese, Cottage cheese |
| Group 4  Ultra-processed foods | Beef Burger, Bacon, Ham, Corned Beef, Sausage, Savory Pies, Liver, Fried Fish, Fish Fingers, White Bread, Brown Bread, Whole Meal Bread, Crisp Bread, Cream Cracker, Cereals, Chips, Lasagna, Pizza, Quiche, Mayonnaise, French Dressing, Margarine (Hard, Polyunsaturated, Soft), Low Fat Spread, Sweet Biscuits, Cakes, Buns, Tarts, Milk Pudding, Sponge Puddings, Ice Cream, Chocolates, Sweets, Crisps, Sauce, Ketchup, Pickle, Marmite, Jam, Coffee Whitener, Hot Chocolate, Horlicks, Soft Drinks, Low-Caloric Soft Drinks, Fruit Squash, Tofu, Soy Meat, Soya Milk, Coleslaw, Baked Beans, Meat Soup, Vegetable Soup, Spirits, Liqueurs. |

^a^All the food items and beverages from 127 food items FFQ were classified using Nova classification system[1,2]

Group 1: Unprocessed or Minimally Processed Foods (MPF)

Foods that are consumed in their natural state or have undergone minimal processing to preserve their freshness, such as washing, peeling, or freezing, without the addition of sugar, salt, oil, or other food additives. Examples include fresh fruits and vegetables.

Group 2: Processed Culinary Ingredients (PCI)

Substances extracted from natural foods or obtained from nature, used to prepare, season, or cook foods. These ingredients are typically not consumed on their own. Examples include oils, butter, sugar, and salt.

Group 3: Processed Foods (PF)

Foods created by combining Group 1 foods with Group 2 ingredients through methods like cooking, canning, or fermentation. These foods usually involve limited processing and contain few added ingredients. Examples include canned vegetables, fresh bread, and cheese.

Group 4: Ultra-Processed Foods (UPF)

Food products that undergo extensive industrial processing and include added artificial flavours, colours, emulsifiers, preservatives, and other additives to enhance taste, texture, or shelf life. Examples include carbonated drinks, ready-to-eat instant meals, and margarine.

S3 Table. ICD-9 and ICD-10 Codes for Cardiovascular Diseases, Coronary Heart Diseases, Type II Diabetes, Mortality Outcomes, and All-Cause Mortality

|  | **Cardiovascular diseases (CVD)** |
| --- | --- |
| ICD 9 | Stroke (430,431,434,436)  Myocardial infarction (410)  Definite angina (410)  Peripheral artery disease (250.7, 440.2, 440.4, 443.8-9, 444.2, 444.81) |
| ICD 10 | Stroke (I60, I61, I63-64)  Myocardial infarction (I21)  Definite angina (I20)  Heart failure (I50)  Peripheral artery disease (I70.2, I73.3, I73.9, I74.3-5, E10.5, E115, E12.5, E13.5 and E14.5) |
|  | **Coronary heart diseases (CHD)** |
| ICD 9 | 410-414 |
| ICD 10 | I20-I25 |
| ICD | I100-I99 |
|  | **Type II diabetes (T2D)** |
| ICD 9 | 250 |
| ICD 10 | E11 |
|  | **CHD mortality** |
| ICD 9 | 410-414 |
| ICD 10 | I20-I25 |
|  | **All-cause mortality** |
| ICD 10 | IHD (I20-I25)  Stroke (I60-I61, I63-I64)  All vascular diseases (I100-I99, R96)  Respiratory (J00-J99)  Cancer (C00-C97)  Non-vascular disease (All except I100-I99 and R96) |

Abbreviations: ICD= International Classification of Diseases, IHD= ischemic heart disease.

S4 Table. Distribution of variables with missing data before and after multiple imputation^a^

| Characteristics | Before Imputed participants | Imputed participants | Number (%) with missing data |
| --- | --- | --- | --- |
| Marital status  Yes  No | 5216(75.4)  1700 (24.6) | 5369 (75.2)  1769 (24.8) | 222(3.1) |
| Family history of CMD  Yes  No | 670 (10.6)  6302 (88.3) | 691 (9.7)  6447 (90.3) | 166 (2.3) |
| Family history of Cancer  Yes  No | 3100 (43.4)  2673 (37.4) | 3689 (51.7)  3449 (48.3) | 1365 (19) |
| Education  < secondary  Secondary  University | 2572 (41.5)  1246 (22.7)  1961 (35.7) | 2636 (36.9)  1919 (26.9)  2583 (36.2) | 1459 (20.4) |
| Employment grade  Administrative  Prof/executive  Clerical/ support | 790 (12.7)  2611(42.1)  2806 (45.2) | 818 (11.4)  3130 (43.8)  3190 (4.7) | 931(13.0) |
| Physical behaviour  Inactive  Active  Moderately active | 415 (6.6)  5456 (86.1)  466 (7.4) | 385(5.4)  6319(88.5)  434(6.1) | 801(11.2) |
| Smoking  Never smoking  Ex-smoker  Current smoker | 2989 (45.8)  3092 (47.3)  449 (7.6) | 3444 (48.2)  3140 (44.0)  554(7.8) | 608 (8.5) |
| Alcohol consumption  Abstainers  Moderate drinkers  Heavy drinkers | 2255 (36.0)  1675 (26.7)  2332 (30.0) | 2636 (36.9)  1919 (26.9)  2583 (36.2) | 875 (12.3) |
| BMI (kg/m^2)^ | 26.4 (4.2) | 26.6 (29.5) | 568 (10.8) |
| Hypertension  Yes  No | 1467 (21.9)  5213 (78.1) | 1787 (25.0)  5351 (75.0) | 458 (6.4) |
| Type II diabetes  Yes  No | 172 (2.6)  6523 (97.4) | 245 (3.4)  6893 (96.5) | 243 (3.4) |
| Dyslipidaemia  Yes  No | 1724 (27.1)  4634 (72.9) | 1903 (26.7)  5235 (73.3) | 600 (8.4) |
| Sodium intake (mg/day) | 2634.0(957) | 2626 (947) | 326 (4.6) |
| Sugar intake (g/day) | 133.2(51.0) | 132 (48.9) | 476 (6.7) |
| Fat intake (g/day) | 77.7 (30.7) | 76.9 (29.5) | 433 (6.1) |

Abbreviations: CMD, cardiometabolic diseases; BMI, body mass index.

^a^Values are mean (standard deviation) or counts (percentages) as indicated

S5 Table. Model search process for Ultra-processed food Trajectories (n=7,318), UK Whitehall II study, 1991/1994-2002/2004

| Number of groups | BIC ^a^ | AIC^b^ | Log- likelihood | Group percentage (%) | Average Posterior probability Assignment (APPA) | Entropy |
| --- | --- | --- | --- | --- | --- | --- |
| 1 |  |  |  |  |  |  |
| 2 | -76885.3 | -76921.5 | -76805.5 | 64.6%  35.4% | 0.87  0.87 | 0.572 |
| 3^c^ | -76515.8 | -76408.2 | -76381.2 | 60.1%  24.6%  15.3% | 0.81  0.77  0.85 | 0.577 |
| 4 | -76474.7 | -76327.2 | -76290.2 | 35.3%  7.7%  47.7%  9.3% | 0.67  0.69  0.75  0.83 | 0.548 |
| 5 | -76492.9 | -76306.1 | 076258.6 | 7.7%  5.7%  47.7%  30.0%  9.2% | 0.64  0.46  0.72  0.57  0.83 | 0.510 |

^a^ BIC: Bayesian information criterion (with higher values indicating a better fit, and log Bayes Factors >10 as strong evidence)

^b^ AIC: Akaike Information Criterion

^c^Although BIC was higher for the 4-group model, we rejected this model because the difference in BIC was fairly small compared to the 3-group model. Furthermore, additional groups largely replicated the patterns of the 3-group model but with smaller group sizes. We therefore selected the 3- group model as the most parsimonious description of the longitudinal patterns in the data.

S6 Table. Determining the Highest Model Function of the 3 Ultra-processed food trajectory groups (n=7,138)

| Trajectory  Shapes ^a^ | BIC ^b^ | Group percentage（%） | P value | APPA | OCC | Entropy |
| --- | --- | --- | --- | --- | --- | --- |
| 2 2 2 | -76494.2 | 15.2  25.5  59.3 | <0.001  <0.001  <0.001 | 0.84  0.78  0.80 | 22.8  10.5  3.3 | 0.574 |
| 2 2 1 | -76487.8 | 55.6  25.3  14.9 | <0.001  <0.001  <0.001 | 0.80  0.77  0.84 | 10.4  3.3  23.4 | 0.576 |
| 2 1 1 | -76495.2 | 25.8  55.2  19.0 | <0.05  <0.05  <0.05 | 0.78  0.80  0.85 | 3.24  10.8  22.7 | 0.573 |

**Abbreviations:** UPF: ultra-processed food; BIC: Bayesian information criterion; OCC:

odds of correct classification

APPA: Average posterior probability assignment

^a^Trajectory shapes of the best fit model according to a given number of shapes: 1= linear;

2= quadratic; 3=cubic

^b^ BIC: Bayesian information criterion (with higher values indicating a better fit, and log

Bayes Factors >10 as strong evidence)

S7 Table. Sensitivity analysis for association between UPF trajectories and risk of CVD, CHD and its subsequent all-cause mortality restricting to participants with all 3 UPF measures (N=4050)^a^

| Outcomes | Low UPF intake | Moderate UPF intake | High UPF intake |
| --- | --- | --- | --- |
| No. of participants | 865 | 2543 | 642 |
| No. of CVD cases | 177 | 468 | 122 |
| CVD HR (95% CI) | Reference | 1.07 (0.87-1.32) | 1.25(1.08-2.26) |
| No. of CHD cases | 144 | 395 | 100 |
| CHD HR (95%CI) | Reference | 1.05 (0.79-1.39) | 1.31 (1.16-3.18) |
| No. CVD deaths | 39 | 102 | 23 |
| CVD mortality HR (95% CI) | Reference | 2.19(0.48-4.25) | 1.27(0.17-3.39) |
| No. CHD deaths | 15 | 55 | 12 |
| CHD mortality HR (95% CI) | Reference | 1.21 (0.60-2.44) | 1.18 (0.69-2.83) |
| No. deaths (all-cause) | 179 | 443 | 115 |
| All- cause mortality HR (95% CI) | Reference | 1.10(0.89-1.36) | 1.01(0.99-1.39) |

**Abbreviations**: HR = CI= confidence interval, Ref= reference, CVD= cardiovascular disease, CHD= coronary heart disease

^a^ Adjusted for the same covariates listed in Figure 2 Model 5

S8 Table. Sensitivity analysis for association between UPF trajectories and risk of CVD, CHD and its subsequent all-cause mortality for complete case data only(N=3735)^a^

| Outcomes | Low UPF intake | Moderate UPF intake | High UPF intake |
| --- | --- | --- | --- |
| No. of participants | 870 | 2276 | 578 |
| No. of CVD cases | 166 | 421 | 115 |
| CVD HR (95%CI) | Reference | 1.06 (0.44-1.32) | 1.20(1.03-2.26) |
| No. of CHD cases | 143 | 350 | 92 |
| CHD HR (95%CI) | Reference | 1.05 (0.44-2.59) | 1.33(1.12-2.57) |
| No. CVD deaths | 34 | 89 | 24 |
| CVD mortality HR (95% CI) | Reference | 1.12(0.89-1.40) | 0.98(0.71-1.14) |
| No. CHD deaths | 15 | 48 | 11 |
| CHD mortality HR (95% CI) | Reference | 1.21 (0.60-2.44) | 0.94 (0.46-2.91) |
| No. deaths (all-cause) | 172 | 395 | 109 |
| All- cause mortality HR (95% CI) | Reference | 1.13(0.83-1.54) | 1.01(0.74-1.40) |

**Abbreviations**: CVD: cardiovascular disease, CHD: coronary heart disease, HR: Hazard ratio, CI: confidence interval, Ref: reference, CVD: cardiovascular disease; CHD: coronary heart disease

^a^ Adjusted for the same covariates listed in Figure 2 and Figure 3 Model 5

S9 Table. Sensitivity analysis for association between 11-year change in UPF intake between phase 3 (1991/1994) and phase 7 (2002/2004) and risk of CVD, CHD and its subsequent all-cause mortality (N=7,138)^a^

| Outcomes | Low UPF intake^b^ | Low to high UPF intake | High to low UPF intake | Moderate UPF intake^c^ | High UPF intake |
| --- | --- | --- | --- | --- | --- |
| No. of participants | 1145 | 476 | 882 | 2882 | 1753 |
| Mean UPF intake at phase 3(% g/day)^d^ | 13.8± 4.5 | 11.6 ± 2.8 | 28.5 ± 7.4 | 19.0 ± 7.9 | 30.5 ± 7.4 |
| Mean UPF intake at phase 7 (% g/day) | 10.3 ± 3.5 | 34.4 ± 7.2 | 12.5± 3.4 | 19.1± 2.0 | 34.3 ± 9.2 |
| No. of CVD cases | 177 | 33 | 71 | 394 | 453 |
| CVD HR (95%CI) | Reference | 1.07 (0.75-1.53) | 0.96 (0.74-1.25) | 0.94(0.78-1.13) | **1.14 (1.02-1.30)** |
| No. of CHD cases | 136 | 24 | 54 | 293 | 352 |
| CHD HR (95%CI) | Reference | 1.01 (0.66-1.54) | 0.97 (0.71-1.31) | 0.90(0.72-1.11) | **1.18(1.03-1.38)** |
| No. CVD deaths | 42 | 4 | 19 | 101 | 118 |
| CVD mortality HR (95% CI) | Reference | 0.59(0.22-1.60) | 1.10(0.66-1.84) | 0.81(0.21-3.13) | 1.19 (0.90-1.56 |
| No. CHD deaths | 18 | 1 | 12 | 42 | 54 |
| CHD mortality HR (95% CI) | Reference | 0.36 (0.05-2.63) | 1.52 (0.76-3.03) | 0.89(0.49-1.61) | 1.30 (0.86-1.97) |
| No. deaths (all-cause) | 227 | 40 | 87 | 454 | 506 |
| All- cause mortality HR (95% CI) | Reference | 0.89(0.62-1.28) | 1.03 (0.81-1.32) | 0.85(0.71-1.02) | 1.04(0.91-1.18) |

**Abbreviations**: CVD: cardiovascular disease, CHD: coronary heart disease, HR: Hazard ratio, CI: confidence interval, CVD: cardiovascular disease; CHD: coronary heart disease

^a^ Adjusted for sex, ethnicity, education level, family history of CVD and CHD and covariates at phase 3 (1991/1994) and phase 7(2002/2004) including sex, age, marital status, physical activity, smoking, alcohol consumption, socioeconomic status, sodium intake, total sugar intake, total fat intake, energy intake, BMI, hypertension, type 2 diabetes, dyslipidaemia.

^b^ The 33rd and 66th percentiles of UPF intake at phase 3 were used as cut-off points to classify UPF intake at Phase 7, resulting in five groups: low, moderate, high, low-to-high, and high-to-low.

^c^ The "Moderate" group includes participants with UPF intake between the 33rd and 66th percentiles at Phase 7. It comprises those with stable moderate intake as well as individuals transitioning from low to moderate or high to moderate.

^d^ UPF intake at Phase 3 and Phase 7 is expressed as **mean ± standard deviation (SD).** "% g/day" refers to the percentage of total daily food intake in grams.

S1 Fig. Flowchart of sample selection

**
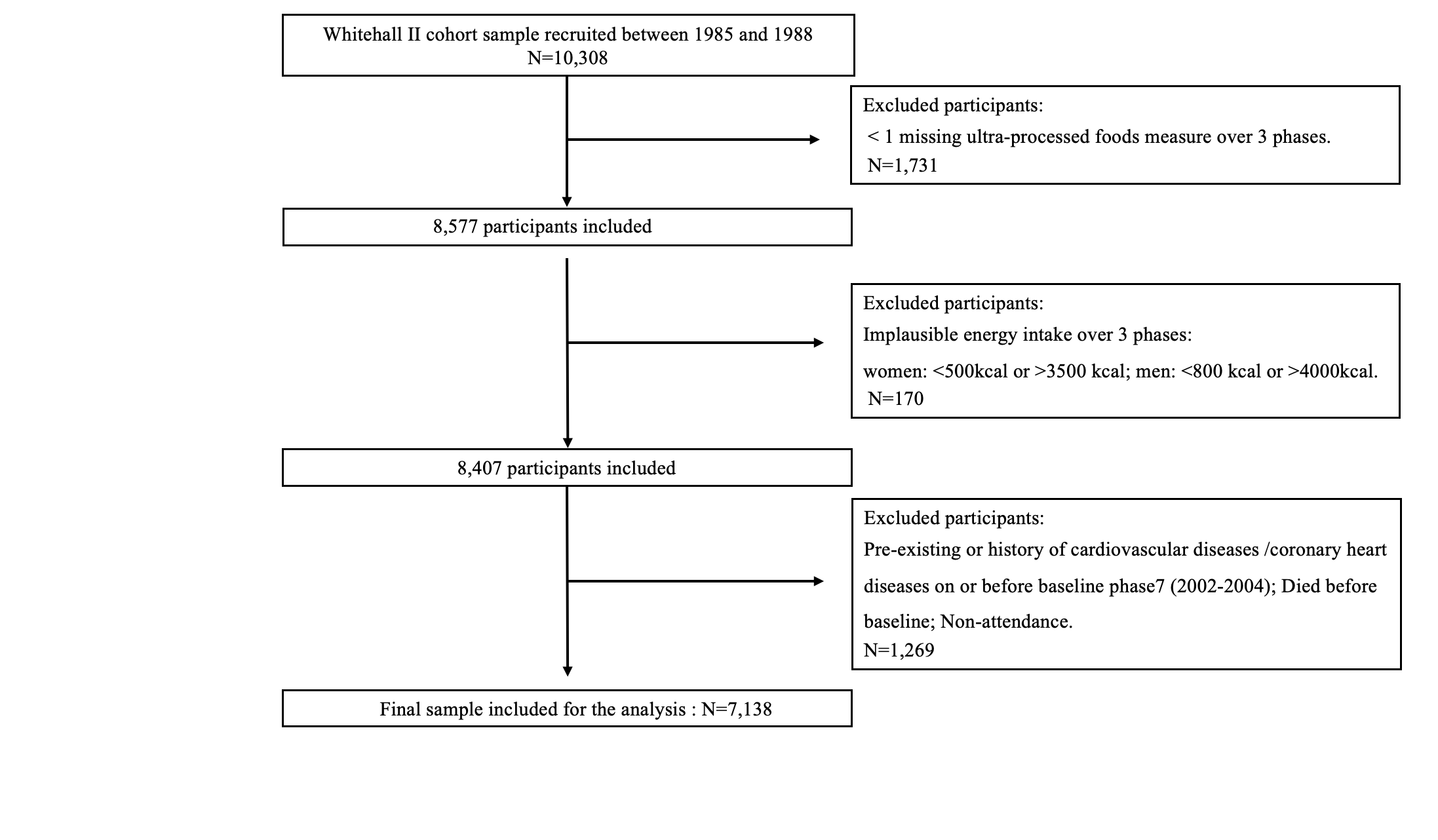
**

S2 Fig. Schoenfeld residuals^a^

Fig S2a). Schoenfeld residuals for incident cardiovascular diseases

**
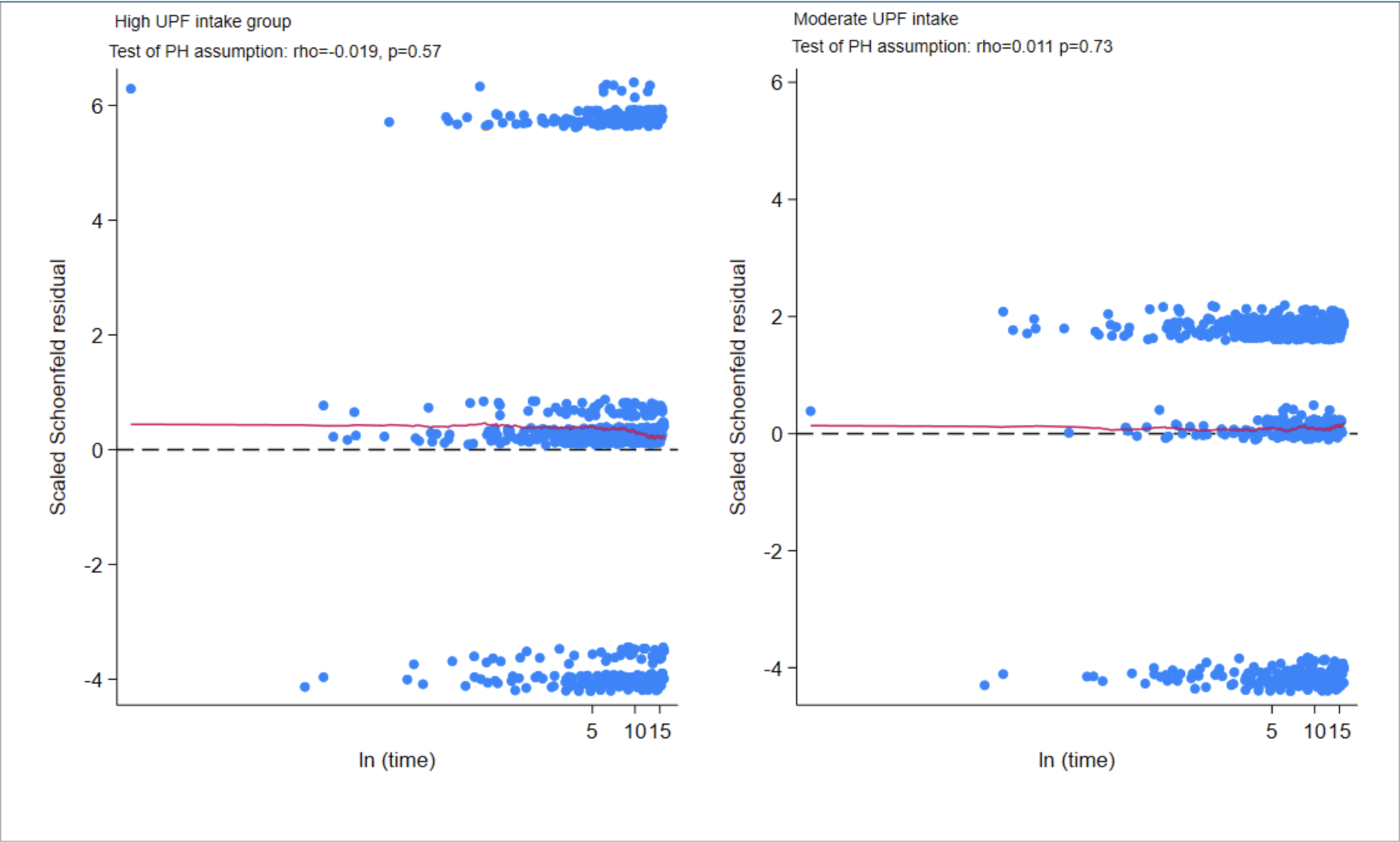
**

^a^ The proportional hazards (PH) assumption was tested using a generalized linear regression of the scaled Schoenfeld residuals on a function of time. A non-zero slope indicates a violation of the PH assumption.

Fig S2b). Schoenfeld residuals for incident coronary heart diseases

**
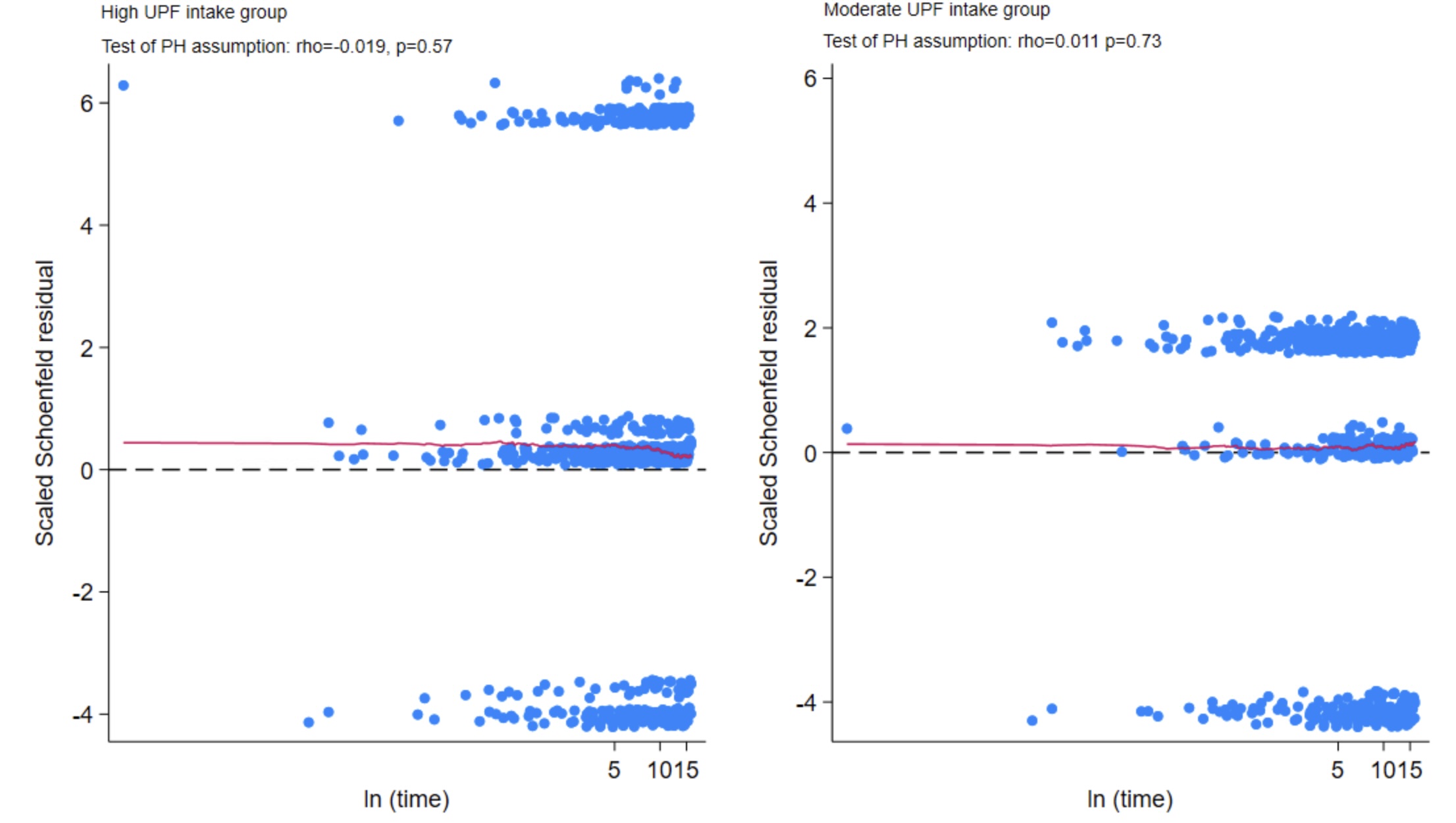
**

Fig S2c). Schoenfeld residuals for cardiovascular diseases mortality

**
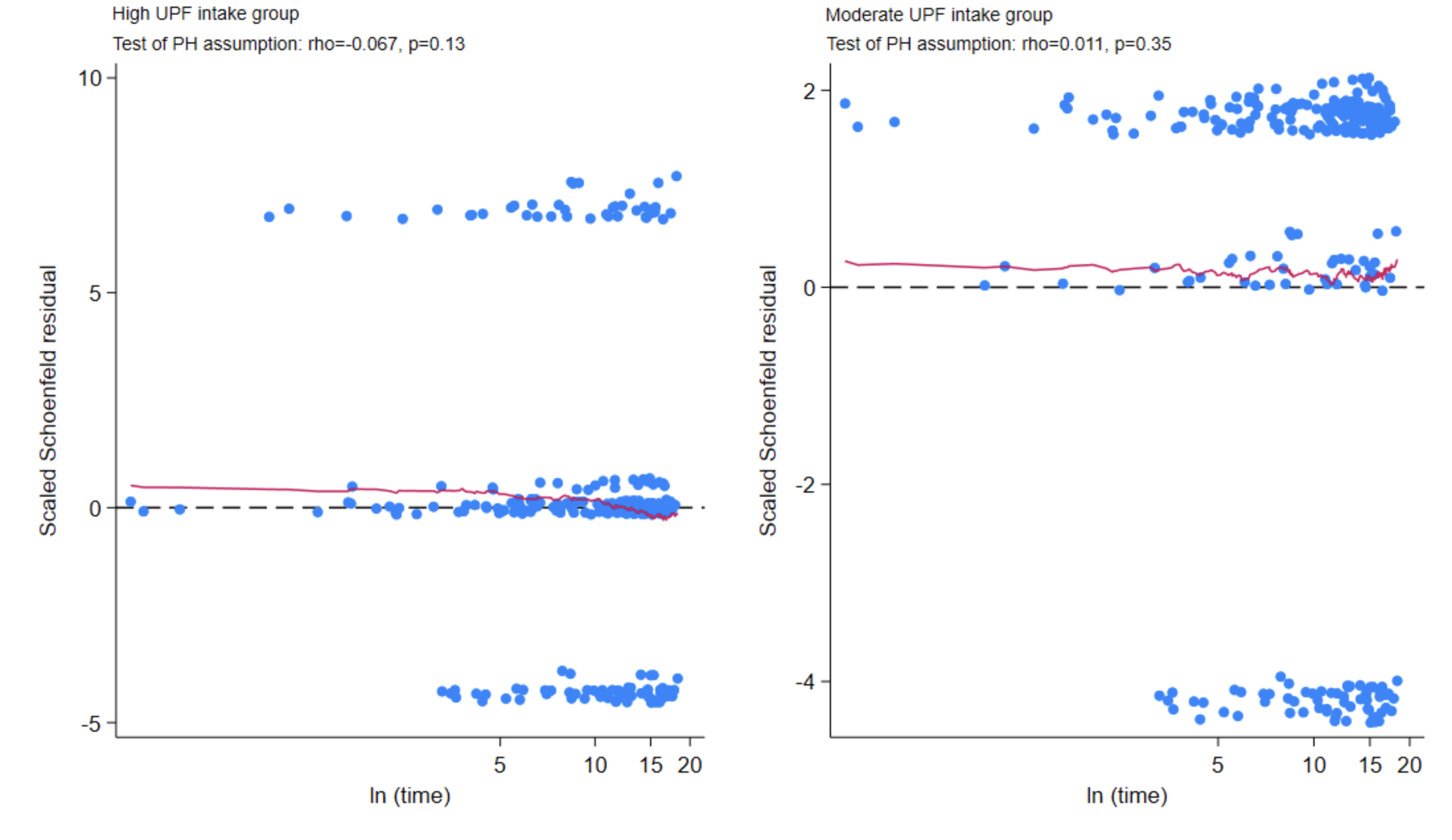
**

Fig S2d). Schoenfeld residuals for coronary heart disease mortality

**
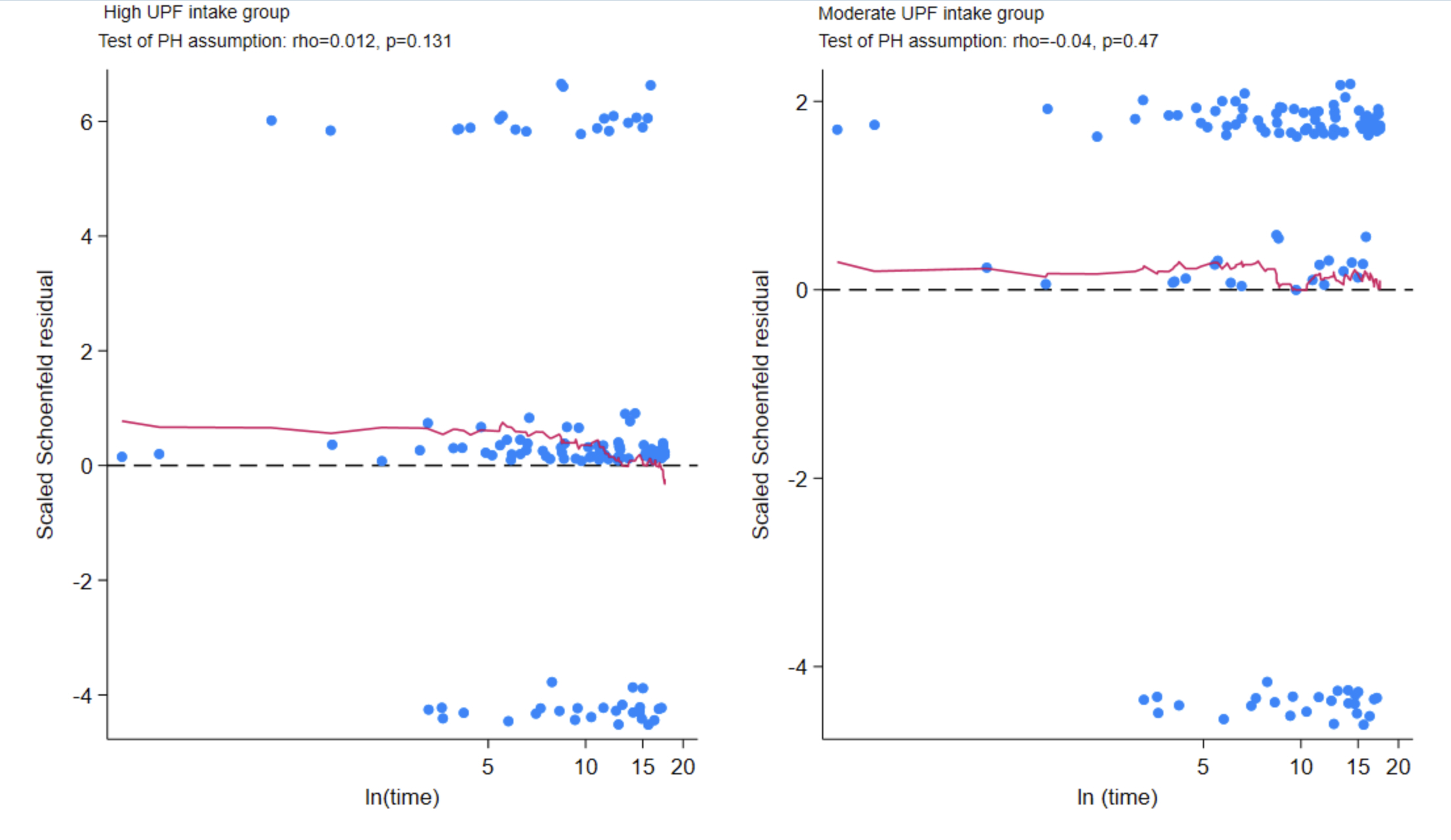
**

Fig S2e). Schoenfeld residuals for all-cause mortality

**
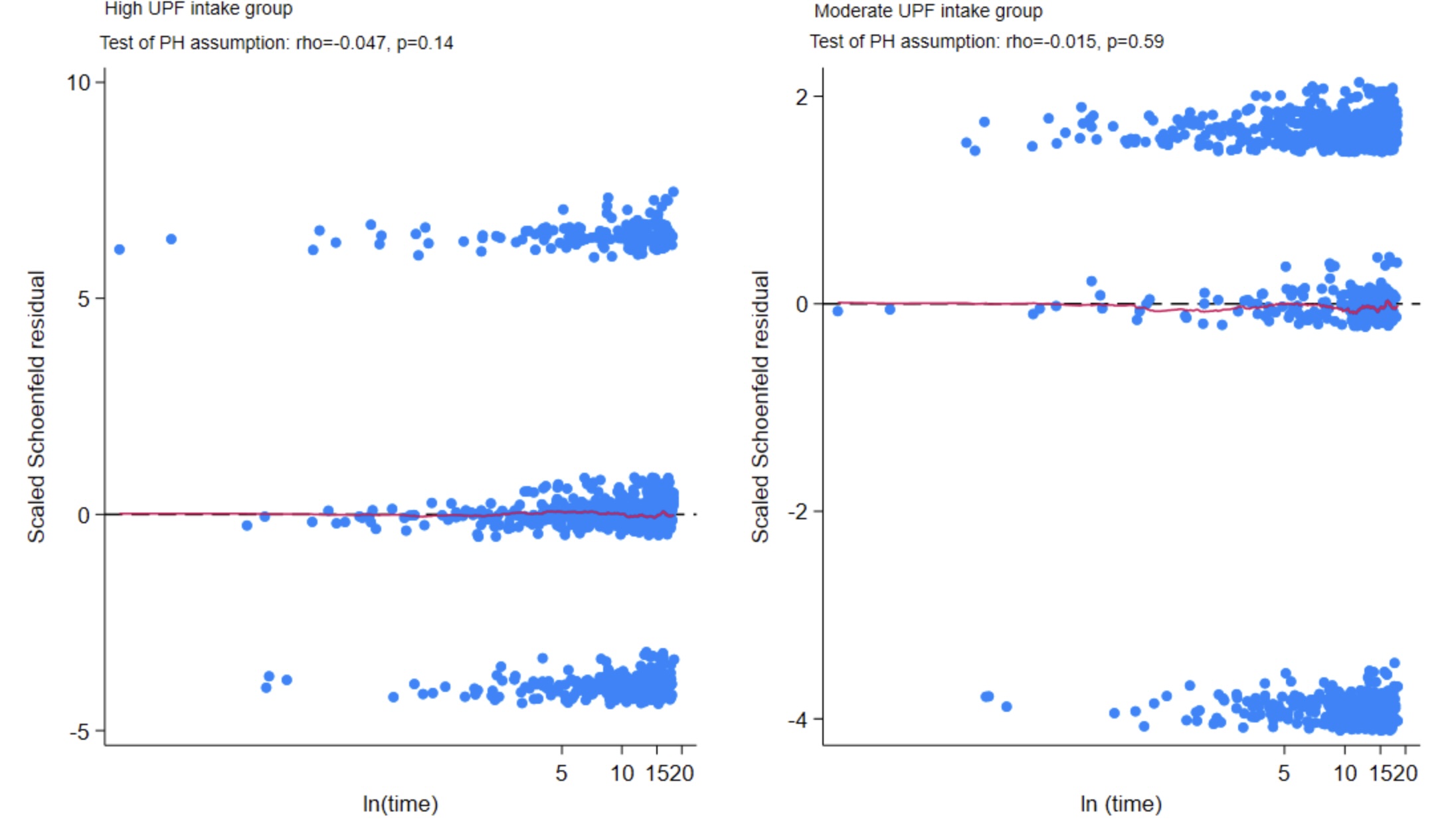
**

Fig S3. Longitudinal trends in UPF intake tertile across phase 3 (1991-1994) to phase 7 (2002-2004)^a^

^a^ This figure illustrates participants' transitions between tertiles from Phase 3 to Phase 7. The percentages indicate the proportion of participants who moved from one tertile to another.

**References**

1. Monteiro CA, Cannon G, Levy RB, Moubarac JC, Louzada MLC, Rauber F, et al. Ultra-processed foods: what they are and how to identify them. Public Health Nutr [Internet]. 2019 [cited 2023 Oct 2];22:936–41. Available from: https://www.cambridge.org/core/journals/public-health-nutrition/article/ultraprocessed-foods-what-they-are-and-how-to-identify-them/E6D744D714B1FF09D5BCA3E74D53A185

2. Monteiro CA, Cannon G, Lawrence M, Laura Da Costa Louzada M, Machado PP. Ultra-processed foods, diet quality, and health using the NOVA classification system Prepared by. [cited 2023 Dec 22]; Available from: http://www.wipo.int/amc/en/mediation/rules
